# Process‐Informed Subsampling Improves Subseasonal Rainfall Forecasts in Central America

**DOI:** 10.1029/2023GL105891

**Published:** 2024-01-05

**Authors:** Katherine M. Kowal, Louise J. Slater, Sihan Li, Timo Kelder, Kyle J. C. Hall, Simon Moulds, Alan A. García‐López, Christian Birkel

**Affiliations:** ^1^ Department of Geography and the Environment University of Oxford Oxford UK; ^2^ Department of Geography University of Sheffield Sheffield UK; ^3^ Climate Adaptation Services Bussum The Netherlands; ^4^ National Oceanic and Atmospheric Administration (NOAA) Physical Sciences Laboratory Boulder CO USA; ^5^ Cooperative Institute for Research in Environmental Sciences NOAA and University of Colorado Boulder Boulder CO USA; ^6^ School of GeoSciences University of Edinburgh Edinburgh UK; ^7^ Department of Earth and Environmental Sciences Columbia University New York NY USA; ^8^ Department of Geography University of Costa Rica San Jose Costa Rica

**Keywords:** rainfall, forecast, Central America, subseasonal, extreme weather, ensemble

## Abstract

Subseasonal rainfall forecast skill is critical to support preparedness for hydrometeorological extremes. We assess how a process‐informed evaluation, which subsamples forecasting model members based on their ability to represent potential predictors of rainfall, can improve monthly rainfall forecasts within Central America in the following month, using Costa Rica and Guatemala as test cases. We generate a constrained ensemble mean by subsampling 130 members from five dynamic forecasting models in the C3S multimodel ensemble based on their representation of both (a) zonal wind direction and (b) Pacific and Atlantic sea surface temperatures (SSTs), at the time of initialization. Our results show in multiple months and locations increased mean squared error skill by 0.4 and improved detection rates of rainfall extremes. This method is transferrable to other regions driven by slowly‐changing processes. Process‐informed subsampling is successful because it identifies members that fail to represent the entire rainfall distribution when wind/SST error increases.

## Key Points

- Subsampling members using sea surface temperatures and zonal wind improves subseasonal ensemble rainfall forecasts in Central America
- In multiple months and locations mean squared error skill increases by 0.4 and extreme rainfall skill improves by 0.5 (Heidke skill)
- Process‐informed subsampling is useful because the models' representation of rainfall degrades as process error increases

## 1 Introduction

Rainfall forecasts can support preparedness for hydrometeorological extremes like droughts and floods (Braman et al., 2013; Domeisen et al., 2022; Merz et al., 2020; White et al., 2022). At the subseasonal scale (14–60 days ahead), early warnings support proactive disaster mitigation activities such as strategies for planting crops (e.g., Flohr et al., 2017, 2018) and transporting resources to higher ground (De Perez et al., 2016). Subseasonal forecasts are challenging because after several weeks atmospheric conditions lose most of their memory and large scale oceanic variability often only provides a limited source of skill (Vitart & Robertson, 2018).

Atmospheric oceanic general circulation models (AOGCMs) provide one way to predict rainfall by generating dynamic predictions of the earth system (Bauer et al., 2015; Hagedorn et al., 2005; Stockdale et al., 2010). Several techniques can correct raw AOGCM outputs, often categorized into calibration (Manzanas et al., 2019) and combination methods (Hemri et al., 2020). Combining AOGCMs to generate multi model ensembles (MMEs) has been advantageous in several cases (e.g., Elvidge et al., 2023; Palmer et al., 2004; Wang et al., 2009), but does not always significantly improve forecast skill compared to calibrated single models because model errors in MMEs are often correlated (Weigel et al., 2009). Traditionally, model evaluation is conducted based on aggregated mean error and variance. Such evaluation techniques, however, may obscure the possibility that models can be right for the wrong reasons (Eyring, 2016; Eyring et al., 2019; Nowack et al., 2020).

New approaches have been proposed to improve AOGCM temperature forecast skill in Europe at seasonal to decadal scales, by capitalizing on process differences *within* AOGCM ensembles (e.g., Dobrynin et al., 2018, 2022; Dusterhus, 2020; Smith et al., 2020). Each model is comprised of multiple members, which when initialized, represent a range of “guesses” of current day states to account for observational uncertainty (Balmaseda et al., 2011). Smith et al. (2020), for instance, improved North Atlantic Oscillation (NAO) representation by subsampling decadal predictions from CMIP5‐6 based on each model member's proximity to the multimodel ensemble mean NAO estimate. Process‐informed subsampling approaches like these are useful not only because they can potentially improve forecast skill, but also because they can provide insights into causal mechanisms that affect model skill.

We expand on these ideas and propose a post‐processing technique to help diagnose and improve AOGCM subseasonal rainfall forecasts in real‐time. As subseasonal forecasts sit between weather and seasonal climate forecasting windows, we investigate process representation of both atmospheric and oceanic drivers of rainfall (sea surface temperature (SST) and zonal wind direction). We hypothesize that if through chance, certain model members predict both SST and zonal wind well one to two months ahead, these same members will also have higher rainfall skill at two months' lead time. To subsample members in a manner that could be operationally deployed, we identify better performing model members at the point of model initialization. We evaluate the predictions of SST and zonal wind direction, two variables that change more slowly over time, enabling us to use preceding observations as a reference to filter out members whose predictions have become unrealistic. We then subsample the ensemble to include only the top performing members, thereby improving the ensemble mean rainfall forecast.

We test our process‐informed subsampling method in Central America, a region in need of further MME optimization where AOGCM skill varies across locations, times of year, and lead times (e.g., Carrão et al., 2018; Hidalgo & Alfaro, 2012, 2015; Kowal et al., 2021, 2023; Maldonado, Alfaro, Amador, & Rutgersson, 2018; Maldonado, Alfaro, & Hidalgo, 2018). Central America presents prediction challenges in part due to the complex interaction of weather patterns originating from both the Pacific and Atlantic oceans and marked topography that moderates moisture transport over the region (Durán‐Quesada et al., 2017, 2020). Forecasts are therefore unlikely to improve by subsampling ensembles using one process alone. The El Niño Southern Oscillation (ENSO—Trenberth, 1997), for instance, is widely recognized as an important driver of ensemble forecast skill (e.g., Scaife et al., 2019) due to its teleconnections with regional rainfall (Durán‐Quesada et al., 2020). ENSO alone, however, cannot always explain regional rainfall deficits (Muñoz‐Jiménez et al., 2019).

**Figure 1:**
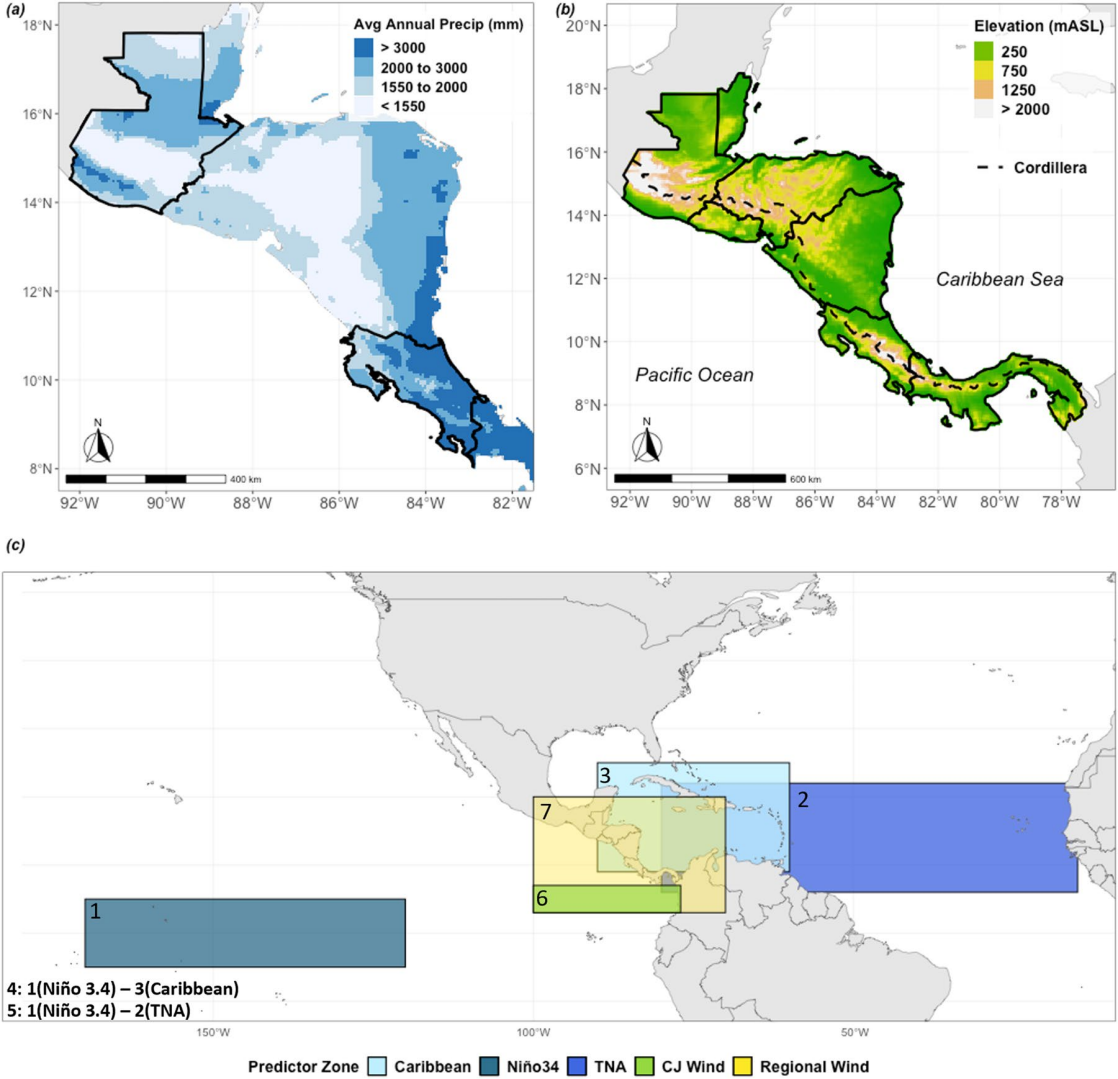
Illustration of Central American climate and tested predictor zones. (a) Total annual rainfall averaged over 1993–2016 using CHIRPS 0.25’ (Funk et al., 2014) monthly rainfall data with Guatemala and Costa Rica outlined in black. (b) Elevation in Central America including the Cordillera. (c) Predictor locations used to test member performance plotted spatially (numbered as labeled in text with SST predictor zones plotted behind country outlines and wind predictor zones plotted in front). Zones include the Caribbean Sea (90W, 60W, 9N, 25N) which includes the core zone for moisture transport illustrated in Durán‐Quesada et al. (2017), Niño 3.4 (170W, 120W, 5N, 5S Trenberth, 1997), and TNA (80W, 15W, 6N, 22N Enfield & Alfaro, 1999); and wind direction over the CJ (100W, 77W, 3N, 7N Poveda & Mesa, 1999) and a broader “Regional Wind” zone that circumscribes the eastern Pacific, core CJ and CLLJ locations (100W, 70W, 3N, 20N).

Here, we subsample ensemble members based on their representation of multiple key regional rainfall‐driving mechanisms, and examine whether our method improves subseasonal rainfall forecast skill in two distinct subregions: Guatemala and Costa Rica (Figure 1a). We then use this approach to assess which large‐scale rainfall‐generating processes need to be accurately captured by models for subseasonal forecasts to be skillful.

## 2 Data

We generated a MME using members from five AOGCMs (Table S1 in Supporting Information S1) that contribute to the leading European seasonal forecasting system (C3S; Marsh & Penebad, 2016). These models' mean monthly estimates are available at 1° spatial resolution over a 24‐year hindcast period (1993–2016) up to six months ahead with a total of 130 members initialized on the first day of every month. Each model contains SST, wind at 850 hPA, and rainfall; and they are commonly deployed for monthly to seasonal forecasts around the world (e.g., Colman et al., 2020; Mishra et al., 2019; Walker et al., 2019).

We used daily Optimum Interpolated Sea Surface Temperature data (OISSTv2—Huang et al., 2021) from National Oceanic Atmospheric Administration (NOAA) as our observational reference data for the models' SST predictions and ERA5 reanalysis data for wind direction (Hersbach, et al., 2018). We used Climate Hazards Group InfraRed Precipitation with Station 0.25° rainfall data (CHIRPS ‐ Funk et al., 2014) to test the final predictions given CHIRPS has been found to be a reasonable data set within the region (e.g., Arciniega‐Esparza et al., 2022). All variables tested (predictors and rainfall predictands) were converted into standardized anomalies prior to testing (Equation S1 in Text S1 in Supporting Information S1).

## 3 Methodology

To support operational feasibility, we used observations just prior to model initialization as reference data to select optimal members when the forecasts were issued. In this approach, we selected members with the best representation of SST and zonal wind direction at one‐ and two‐month lead times. SST and zonal wind were chosen because they are key drivers of regional rainfall (see 3.1) and typically have greater persistence over other variables. Although the magnitude of windspeed may change instantaneously, the low‐level jet wind direction is often seasonally stable (Figure S1 in Supporting Information S1).

We assessed the usefulness of this subsampling technique on monthly rainfall forecasts in the month after initialization (labeled two‐month lead per C3S naming conventions). For example, to generate a subsample for September rainfall predictions, we extracted all available members from the models initialized on 1 August at two separate lead times: one‐month lead (August) and two‐month lead (September). We selected members that best represented our chosen predictors at one‐ and two‐month lead times using observations averaged over the last two weeks of July (reference period) and used only those top performing members in our final two‐month lead rainfall prediction. Because this method relies on preceding observations to select members at one‐ and two‐month lead times, the method will work better when SST and zonal wind direction change more slowly over our predictor zones.

We tested the subsampling method in May, June, September, and October to see how its ability to constrain ensemble predictions of rainfall varied temporally. The Central American climate is characterized by monthly rainfall variations, separated into a wet and dry season with peak rainfall often in May/June (early wet season) and September/October (late wet season) (Giannini et al., 2000; Magaña et al., 1999; Taylor & Alfaro, 2005). We evaluated the subsampling method over the entirety of Costa Rica and Guatemala respectively as key case studies and considered spatial patterns in subsample skill. Rainfall connections with relevant predictors (3.1) for the Caribbean and Pacific sides of the region, for instance, vary in part due to how the Cordillera mountain range (Figure 1b) interacts with moisture transport from the Caribbean Sea to the Pacific Ocean (Durán‐Quesada et al., 2017; Muñoz‐Jiménez et al., 2019; Taylor & Alfaro, 2005).

### 3.1 Relevant Predictor Zones for Central America

We evaluated seven predictor types with well‐documented connections to regional rainfall (Figure 1c), including (a) Niño 3.4 SSTs (Giannini et al., 2000; Sánchez‐Murillo et al., 2017; Waylen et al., 1994), (b) Tropical North Atlantic (TNA) SSTs (Enfield & Mayer, 1997; Maldonado et al., 2017), and (c) Caribbean SSTs (Durán‐Quesada et al., 2017, 2020). We also evaluated representation of the SST gradient across the isthmus (Alfaro, 2007; Enfield & Alfaro, 1999; Enfield & Mayer, 1997; Hidalgo et al., 2015; Maldonado, Alfaro, & Hidalgo, 2018). We used two different SST gradients as predictors to account for the spatial and temporal variability in the relationship between SST gradients and regional rainfall: (d) the maximum anomaly difference between the Niño 3.4 region and the Caribbean (Wang, 2007), and (e) the maximum anomaly difference between Niño 3.4 and TNA SSTs (Enfield & Alfaro, 1999; Taylor et al., 2002). Because the Caribbean Low Level Jet (CLLJ—Amador, 2008) and Chorro del Occidente Colombiano Jet (CJ ‐ Poveda & Mesa, 1999) are also well‐documented moisture transport mechanisms across Central America (Cook & Vizy, 2010; Hidalgo et al., 2015; Muñoz et al., 2008; Poveda & Mesa, 1999; Wang, 2007), we assessed zonal wind direction (f) using CJ alone, and (g) within a region that circumscribes the core CLLJ, Central America, and the eastern Pacific where the CJ crosses the isthmus (Figure 1c). CLLJ was not used alone as a predictor because there was not a large difference between member representation of the core CLLJ (Text S2 in Supporting Information S1).

### 3.2 Member Performance and Top Performing Subsamples Selection

**Figure 2:**
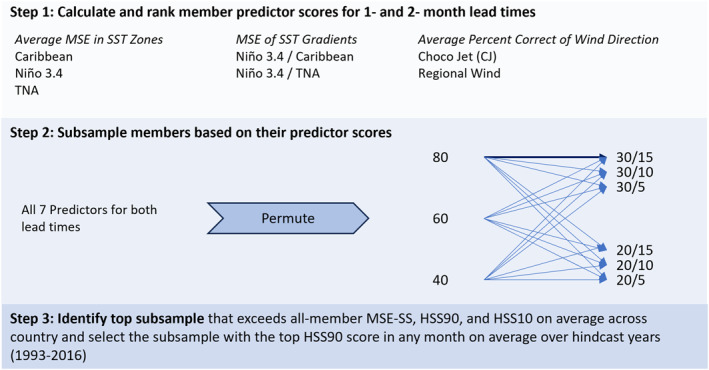
Schematic of method used to estimate how members represent physical processes. Step 1: rank members based on their predictor scores for all seven predictors. Step 2: Subsample members based on their predictor scores using permutations (order does not matter) of predictors. In this subsampling step, filter size options include 80/60/40 (filter 1); 30/20 (filter 2); or 15/10/5 (filter 3) members. For example, (bolded arrow), select top 80 members that represent Niño 3.4 SSTs best at two‐month lead, then of those 80 members, select top 30 members that represent TNA SSTs best at one‐month lead, then of those 30 members select top 15 members that represent CJ wind direction best at one‐month lead. Do this for every possible order of predictors across the filter size options. Step 3: evaluate subsample skill scores using MSE‐SS, HSS90, and HSS10 and identify top subsamples that scored as well as the all‐member ensemble mean across all metrics on average over a country. Although many subsamples meet these criteria, for illustration purposes in the results section, top subsample is selected using HSS90.

We processed all model data with the XCast Python package (Hall & Acharya, 2022; Text S3 in Supporting Information S1) and evaluated predictor estimates from the raw 130‐member ensemble at 1° spatial resolution. We regridded the 130‐member ensemble rainfall predictions to 0.25° resolution using bilinear interpolation prior to subsampling. The 130 estimates of each predictor were ranked (Figure 2, step 1) using the average mean squared error (MSE; Equation S2 in Text S1 in Supporting Information S1) across the predictor zone for SST (or calculating one MSE estimate for the gradients with no spatial field). The average Percent Correct Score (Equation S3 in Text S1 in Supporting Information S1) was used to rank the members' predictions of zonal wind direction within a predictor zone (e.g., CJ). We filtered members based on their relative performance to one another (e.g., top 10) to keep ensemble size consistent.

The “best” subsample is application dependent. Here we showcase a few subsamples that performed well on average over both countries using deterministic criteria over 1993–2016. We used multiple metrics to evaluate rainfall skill of the subsampled means against the all‐member mean, including the Mean Squared Error Skill Score (MSE‐SS—Deque, 2012, Equation S4 in Text S1 in Supporting Information S1) to assess performance across the entire rainfall distribution and the Heidke Skill Score (HSS ‐ Heidke, 1926, Equation S5 in Text S1 in Supporting Information S1) to measure ensemble mean detection rate of low (high) rainfall extremes below the 10th (above the 90th) percentile of monthly anomalies.

We generated subsamples using our ranked predictor scores to select members for permutations of one, two, and three predictors, with several filter size options (Figure 2 step 2). Generating thousands of subsamples across a range of predictor permutations enabled us to compare subsamples that prioritized more well‐rounded skill (using multiple predictors to select members) against subsamples that prioritized representation of a particular predictor. We identified a subset of better performing subsamples in which rainfall predictions outperformed the all‐member mean using all deterministic scores (MSESS, HSS90, and HSS10) in any month (May, June, September, or October) on average over a country for the entire assessed period (Figure 2 step 3). We further explored the subsamples' sensitivity to filter size. Many filter sizes resulted in skillful subsamples (Text S4 in Supporting Information S1), and when filter size did matter, it often indicated the relative importance of different predictors (Figure S2 in Supporting Information S1).

This selection procedure identifies subsamples that on average maintain better skill across the analyzed years. Because of the interannual variability in ensemble forecast skill, we performed a sensitivity test to evaluate the robustness of the approach (Text S5 in Supporting Information S1). Although the skill of the all‐member and subsampled ensembles changes interannually (Figure S3 in Supporting Information S1), randomly dropping five years from the analysis does not significantly change the difference in skill between the all‐member mean and subsampled mean in almost any case tested (Figure S4 in Supporting Information S1). To better understand what happens to the models' rainfall predictions when predictor error increases, we also examined the correlation between the model rainfall predictions and the estimates of the seven predictors for each month analyzed.

## 4 Results and Discussion

### 4.1 Top Subsamples Align With Key Processes That Drive Regional Rainfall

The “best” subsample depends on the application (e.g., many subsamples detect high rainfall extremes well but fail to consistently detect low rainfall extremes). Our results below summarize subsamples that outperform the all‐member mean across all three metrics (MSESS, HSS90, and HSS10) on average within a country. Several subsamples outperform the all‐member mean across all metrics for a given month, but the ones highlighted here have the top HSS90 skill for detections of high rainfall extremes (Table 1).

**Table 1:** Summary of Top Subsamples Identified by Country and Months Where Both Countries Have Subsamples That Outperformed the All‐Member Mean on Average Across All Metrics

Predictand month	Filter step	Costa Rica		Guatemala	
		Predictors (filter size)	Average [worst, best] change in predictor score	Predictors (filter size)	Average [worst, best] change in predictor score
May	1	SSTs in Caribbean	**MSE: −0.24**	SSTs in TNA	**MSE: −0.24**
		1‐month lead (60)	[−0.11, −0.59]	1‐month lead **(10)**	[−0.12, −0.77]
	2	CJ Wind	**%Correct: 21%**		
		2‐month lead (20)	[10%, 33%]		
	3	SST Gradient Niño3.4/TNA	**MSE: −0.63**		
		2‐month lead **(5)**	[−0.17, −2.48]		
September	1	SSTs in Niño 3.4	**MSE: −0.02**	SSTs in Niño 3.4	**MSE: −0.02**
		1‐month lead (80)	[0.01, −0.06]	1‐month lead (80)	[0.01, −0.06]
	2	SSTs in Niño 3.4	**MSE: 0.06**	SSTs in Niño 3.4	**MSE: 0.05**
		2‐month lead (30)	[0.16, 0.01]	2‐month lead (30)	[0.16,−0.03]
	3	Regional Wind	**%Correct: 3%**	SST Gradient Niño3.4/Caribbean	**MSE: −0.54**
		1‐month lead **(15)**	[−1%, 6%]	2‐month lead **(10)**	[−0.20, −1.12]
October	1	SSTs in Niño 3.4	**MSE: −0.09**	CJ Wind	**%Correct: 12%**
		2‐month lead (40)	[−0.04, −0.19]	1‐month lead (60)	[3%, 30%]
	2	SST Gradient Niño3.4/Caribbean	**MSE: 0.28**	SST Gradient Niño3.4/TNA	**MSE: 0.70**
		2‐month lead (20)	[0.70, −0.12]	2‐month lead (20)	[1.63, 0.19]
	3	SST Gradient Niño3.4/Caribbean	**MSE: −0.10**	CJ Wind	**%Correct: 5%**
		1‐month lead **(15)**	[0.41, −0.49]	2‐month lead **(15)**	[−12%, 10%]

*Note*. The top subsamples for each month are listed next to the order of operations used to filter each predictor with number of members selected in each step in parentheses and the average [worst, best] change in predictor scores over 1993–2016 between the final subsample and the all‐member mean. Improvements in predictor score are reported in terms of MSE for SST zones and SST gradients and Percent Correct for wind direction, as calculated for ranking members. Decreases in MSE (more negative) and increases in Percent Correct scores show improvements in predictor score. Final ensemble sizes and average changes in predictor errors are bolded.

Subsamples that use three predictors to filter members tend to outperform subsamples with fewer predictors, except when using TNA alone in May over Guatemala (Table 1 row 1 right column). We found no subsamples in June that outperformed the all‐member mean in Costa Rica across all metrics, while the ten‐member TNA subsample that performed best in Guatemala in May still performed well in June. More limited success in June may be related to the stronger influence of atmospheric drivers that affect moisture transport (Durán‐Quesada et al., 2017), or higher predictor variability, as the lagged observations were poorly correlated with predicted months in that period (Figure S1 in Supporting Information S1). While our method may constrain error that occurs in a large ensemble, any subsampling procedure is also subject to uncertainties in observational data sets. The usefulness of the method depends on these data sets being close enough approximations of “real‐world” conditions to help filter out unrealistic members.

Using process‐informed criteria to select members from a large ensemble can help diagnose why model skill may vary (Eyring et al., 2019; Nowack et al., 2020). The top subsamples often relate to seasonal drivers of rainfall. In September, for instance, top subsamples primarily rely on Niño 3.4 SSTs, when ENSO drives rainfall more strongly, while in the early wet season, Caribbean and TNA SSTs are more important (Table 1 row 1) when the Caribbean and Atlantic have stronger correlations with regional rainfall (Durán‐Quesada et al., 2017; Maldonado et al., 2017; Spence et al., 2004; Taylor et al., 2002). SST Gradients improve subsamples across the wet season, as they are important throughout (Enfield & Alfaro, 1999; Giannini et al., 2000; Hidalgo et al., 2015; Maldonado, Alfaro, & Hidalgo, 2018).

The top subsample over Guatemala in October is less straightforward, as it is filtered primarily using CJ representation. CJ nears peak strength in October as the intertropical convergence zone is displaced northwards (Amador et al., 2016), but this jet primarily affects rainfall south of Guatemala (Durán‐Quesada et al., 2010, 2017). This predictor may indicate related model errors (e.g., if members poorly represent CJ, they may also poorly represent other drivers of Guatemalan rainfall in October).

Average predictor representation is not always substantially different between the all‐member mean and the top subsamples. The all‐member mean typically includes members with mixed levels of skill—some with high skill, some with low skill, and some with splintered skill (high in some predictor metrics and low in others). The top subsample sometimes improves representation across all predictors in the analysis (e.g., subsamples in May for both countries), meaning the subsample filters out low‐skill members. In other cases, average process representation of the top subsample is similar to (e.g., in September) or even worse than the all‐member mean (e.g., average MSE of some SST gradient representations in October). In these cases, the top performing subsamples filter out splintered skill members from the all‐member ensemble. In October, for instance, the subsamples select more well‐rounded members that can better represent multiple predictors (e.g., CJ wind and SST gradients) compared to the filtered out splintered skill members that poorly represent certain predictors. Further, no single AOGCM consistently outperforms the others, and model representation within subsamples varies interannually (Text S6 in Supporting Information S1), not necessarily linked to the total number of members per AOGCM (Table S2 in Supporting Information S1).

### 4.2 Subsamples Improve Skill for Entire Rainfall Distribution and the Extreme Tails

**Figure 3:**
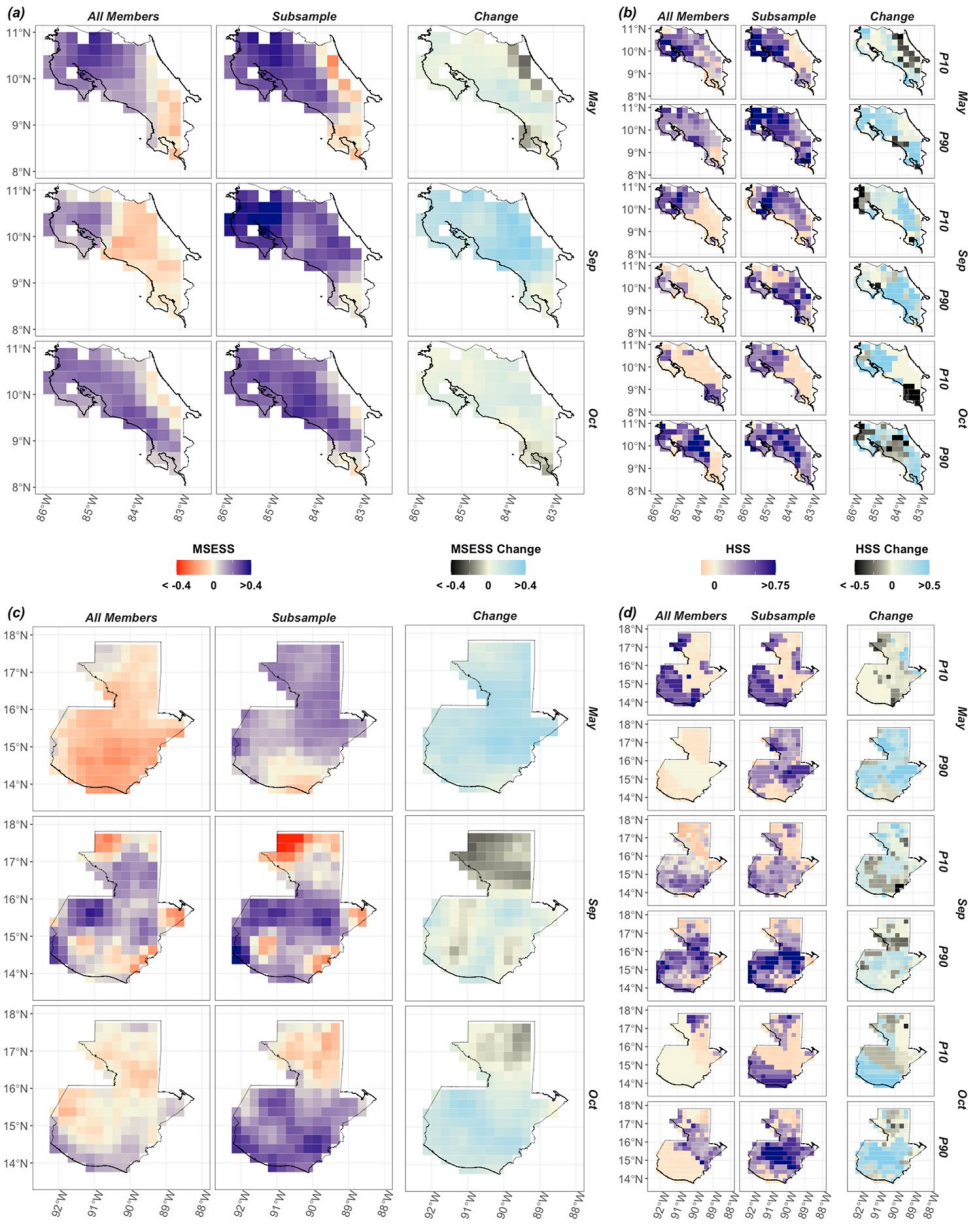
Spatial performance of the method in Costa Rica and Guatemala. (a) Spatial skill of all‐member ensemble (left), top performing subsampled ensemble (center) and difference between the two (right) in Costa Rica. Skill is based on the entire rainfall distribution using MSE‐SS. (b) Spatial skill for detection of low rainfall extremes (P10) and high rainfall extremes (P90) using HSS in Costa Rica, again for all‐member (left), subsample (center), and their difference (right). Panels (c) same as (a) but for Guatemala. Panel (d) same as (b) but for Guatemala. Table 1 summarizes the criteria used to generate the subsamples in each month that were selected for their ability to perform as well as the all‐member mean across all three metrics (MSESS, HSS10, HSS90) and were the top performers for HSS90 in each month as illustrated in Figure 2.

In all three months, the subsamples improve MSE‐SS across most of Costa Rica and Guatemala (Figure 3a/3c), with the largest change in September over Costa Rica (70% locations increase ≥0.2 MSE‐SS, 55% locations increase ≥0.3 HSS90, 33% locations increase ≥0.3 HSS10) and in May over Guatemala (56% locations increase ≥0.2 MSESS, 62% locations increase ≥0.3 HSS90, 5% locations increase ≥0.3 HSS10). The skill of predicting rainfall extremes improves more in some locations (Figure 3b/3d) with >0.5 changes in HSS for P10 and P90 detections. Filtering out members with low skill is clearly beneficial in Guatemala in May, when the top subsample filters members using TNA representation alone (Figure 3c/3d top row). Filtering out splintered members is useful in Costa Rica in September, when the top subsample significantly improves skill (Figure 3a/3b middle row) despite similar average predictor scores as the all‐member mean (Table 1). These results are similar at the raw 1° spatial resolution (Figure S5 in Supporting Information S1).

Improving forecast skill across an entire country using one subsampling configuration is challenging due to nuances in how processes drive rainfall. Some improvements are spatially limited, highlighting locations where predictor relationships with rainfall may diverge. For instance, in May, the top Costa Rican subsample mostly improves skill on the Pacific side of the country (Figure 3a/3b). Pacific rainfall patterns are distinct from Caribbean rainfall often due to interactions between regional rainfall drivers and topography (Durán‐Quesada et al., 2017; Muñoz‐Jiménez et al., 2019). The top subsamples also show limited skill above 16°N in Guatemala in September and October (Figure 3c/3d). This spatial pattern in skill somewhat corresponds to differences between rainier locations in the country that are closer to the Caribbean Sea, and drier locations in Guatemala with rainfall more dominated by the Pacific (Figure 1a), including the Guatemalan Dry Corridor (Gotlieb et al., 2019). This method could therefore benefit from using more locally optimized subsamples (e.g., over Caribbean vs. Pacific rainfall regions in Costa Rica, Dry Corridor in Guatemala).

### 4.3 Models Fail to Represent Full Rainfall Distribution When Process Error Increases

**Figure 4:**
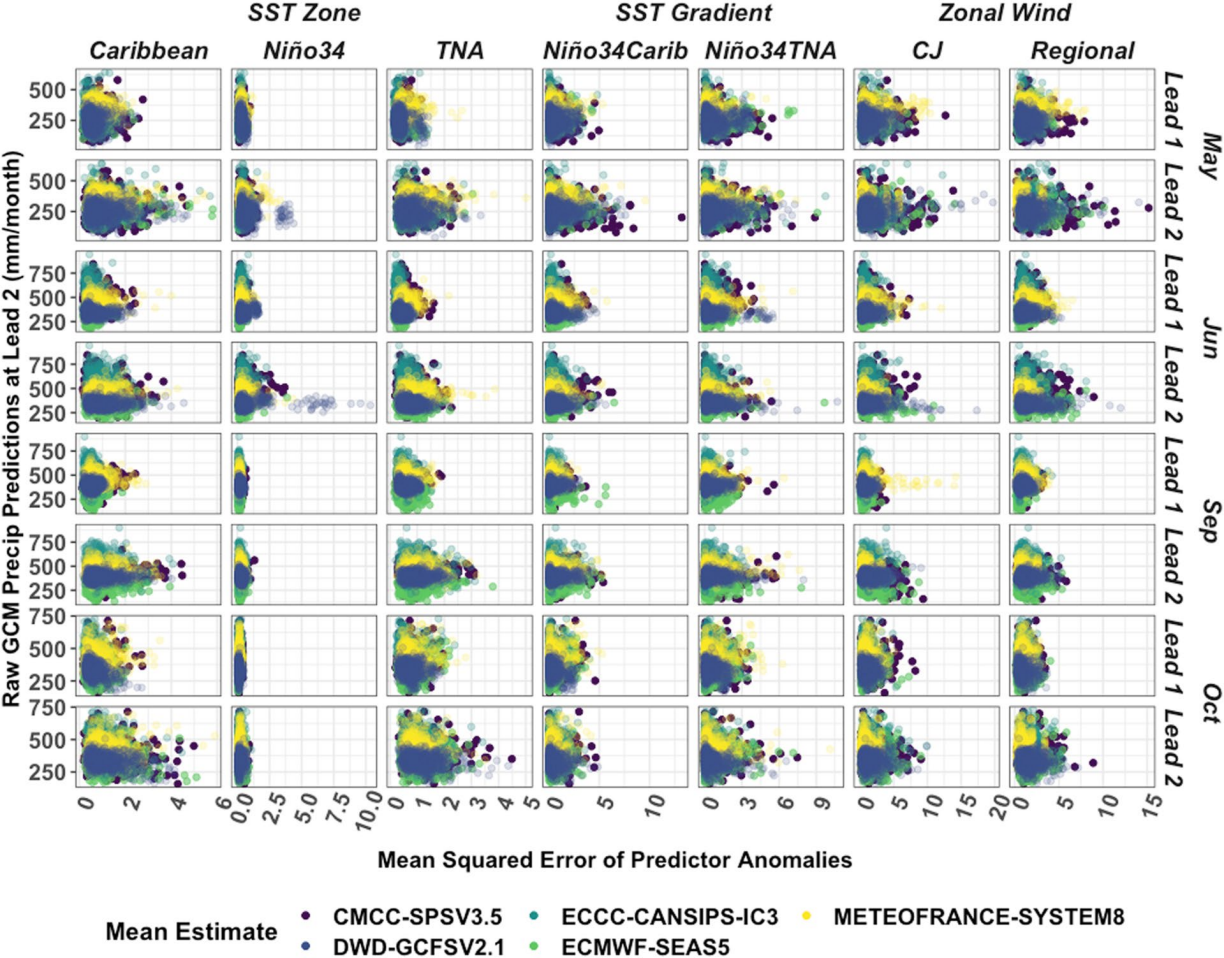
Relationship between the spatial average of the raw precipitation prediction at 2‐month lead (*y*‐axis) over 1993–2016 over each country and the MSE of the predictors (*x*‐axis) for different zones/predictors (columns) grouped by lead time (one month ahead or two months ahead, rows) for different target months (rows), color‐coded by the five models evaluated.

We find when the models struggle to represent certain processes, as seen through estimates of their predictor error using MSE, they are less able to capture the full range of the rainfall distribution (Figure 4). This can be seen in the way the maximum rainfall predictions trend downward (*y*‐axis) as predictor error increases (*x*‐axis) across predictor type (columns) in each month tested (rows). Similarly, an upwards trend in the lowest rainfall predictions is also seen across predictors as error increases. For SST zones like Niño3.4 and for shorter lead times (one‐month lead), the range in error is smaller than other regions at longer lead times (e.g., Caribbean at 2‐month lead), but the slope is steeper, meaning small process errors can have a greater influence on the models' ability to predict rainfall (Figure 4).

Despite some structural differences among the models (e.g., DWD System 2.1 has a smaller rainfall range than the other models, Figure 4), the relationship between process error and the estimated rainfall range is consistent. This consistent cone‐shaped pattern across models showcases the value of the process‐informed subsampling method to support operational forecasting. While a regional forecaster may not have as much familiarity as a model developer with the structural nuances of each model, they can leverage their expertise in regional rainfall drivers to select members that best represent important predictors across ensembles. Some members with low process error may still be selected from a more limited rainfall distribution that is unique to their parent model (e.g., in the case of DWD System 2.1), but by minimizing some key process errors, forecasters can increase the likelihood that a subsample better captures the entire rainfall distribution.

## 5 Conclusions

Subseasonal predictions present a gap between weather and seasonal climate forecasting that could benefit from optimization (Vitart & Robertson, 2018). Our subsampling method presents an opportunity when a forecast is issued (initialized) to select the model members that by chance correctly represent key rainfall predictors to improve rainfall predictions a month ahead. We show the models are less able to capture the full range of the rainfall distribution when SST and zonal wind error increases (Figure 4). This method presents potential for better extreme rainfall preparedness planning by improving extreme rainfall predictions. Process‐informed subsampling may improve MME rainfall skill especially when regional rainfall is driven by slowly‐changing processes like SSTs. Given the interannual variability of key processes, future work could enhance our selection procedure by selecting subsamples based on representation of key processes that are empirically derived from a moving evaluation window. This technique is ideal for constraining regional forecasts based on knowledge of regional rainfall mechanisms and could especially benefit regions where SSTs are a key rainfall driver.

## Conflict of Interest

The authors declare no conflicts of interest relevant to this study.

## Supporting information

Supporting Information S1

## Data Availability

All data is publicly available and free to use. C3S monthly hindcasts of total rainfall, SST, and zonal wind at 850 pressure can be accessed from the Climate Data Store (Copernicus Climate Change Service—CDS, 2018). ERA5 reanalysis data are also freely available at the Climate Data Store (Hersbach et al., 2018). CHIRPS data (Funk et al., 2014) was accessed using the IRI Climate Data Library via the pyCPTv2 software package (Muñoz et al., 2019). OISSTv2 daily SST data (Huang et al., 2021) is hosted by the NOAA Physical Sciences Laboratory. All analysis was processed in XCast (Hall & Acharya, 2022) and visualized in R with ggplot2 and tidyverse (Wickham, 2016; Wickham et al., 2019) as described in Text S3 in Supporting Information S1. Code is available at Kowal (2023).
