# Supplementary material for: Process‐Informed Subsampling Improves Subseasonal Rainfall Forecasts in Central America
Source: Geophys Res Lett. 2024 Jan 5;51(1):e2023GL105891. doi: 10.1029/2023GL105891 (PMC11235057; doi:10.1029/2023GL105891)
Supplement: Supplementary file 1 — Supporting Information S1 [file GRL-51-e2023GL105891-s001.pdf]

Geophysical Research Letters

Supporting Information for

Process-Informed Subsampling Improves Subseasonal Rainfall Forecasts in Central America

Katherine M. Kowal[1*], Louise J. Slater[1], Sihan Li[2], Timo Kelder[3], Kyle J. C. Hall[4,5], Simon Moulds[1,6], Alan A. Garcia-Lopez[7], and Christian Birkel[8]

[1] Department of Geography and the Environment, University of Oxford, Oxford, United Kingdom

[2] Department of Geography, University of Sheffield, Sheffield United Kingdom

[3] Climate Adaptation Services, Bussum, The Netherlands

[4] National Oceanic and Atmospheric Administration (NOAA) Physical Sciences Laboratory, Boulder CO

[5] Cooperative Institute for Research in Environmental Sciences (CIRES), NOAA and University of Colorado Boulder, Boulder CO

[6] School of GeoSciences, University of Edinburgh, Edinburgh, United Kingdom

[7] Department of Earth and Environmental Sciences, Columbia University, New York, NY, United States

[8] Department of Geography, University of Costa Rica, San Jose, Costa Rica

*Corresponding author: Katherine Kowal (katherine.kowal@ouce.ox.ac.uk)

Contents of this file

Text S1, Text S2, Text S3, Text S4, Text S5, Text S6

Figure S1, Figure S2, Figure S3, Figure S4, Figure S5

Table S1, Table S2

Text S1. Formulas used to process and evaluate the ensemble members

The skill scores were applied individually for each grid cell on data that had been converted from raw values into monthly standardized anomalies. Standardized anomalies were calculated monthly across the time series (i.e. months were calculated individually) for each grid cell individually following Equation 1:

$$\boldsymbol{Standardized\ Anomaly} = \frac{\mathbf{x} - \mu(\mathbf{x})}{\sigma(\mathbf{x})} \qquad \text{Equation 1}$$

$x = estimate\ (either\ a\ forecast\ or\ an\ observation)$
$\mu(x) = mean\ of\ estimate\ over\ time\ period\ (e.g.\ 1993 - 2016)$
$\sigma(x) = standard\ deviation\ of estimate\ over\ time\ period\ (e.g.\ 1993 - 2016)$

Predictor representation was scored using mean squared error (MSE, Equation 2) and a Percent Correct Score (Equation 3). MSE is a measure of average accuracy, calculated by adding the squared difference between the observations and the forecasts for every point in time and dividing by the total number of instances (every date). When calculated for an SST Gradient, the MSE score alone was used. When calculated for an SST zone, the average MSE score over the entire grid-box was calculated.

$$\boldsymbol{MSE} = \frac{\mathbf{1}}{\boldsymbol{n}}\sum_{\boldsymbol{i}=\mathbf{1}}^{\boldsymbol{n}}(\hat{\boldsymbol{x}}_{\boldsymbol{i}} - \boldsymbol{x}_{\boldsymbol{i}})^{\mathbf{2}} \qquad \text{Equation 2}$$

$\hat{x} = observation$
$x = forecast$
$n = total\ number\ of\ instances$

Percent Correct Score was calculated for the binary zonal wind direction variables by calculating the number of times the model member predicted wind direction correctly divided by the total number of predictions of wind direction over a grid-box (e.g. CJ).

$$\boldsymbol{Percent\ Correct} = \frac{\boldsymbol{a}}{\boldsymbol{n}} * 100 \qquad \text{Equation 3}$$

$a = correct\ detections$
$n = total\ number\ of\ instances$

The formulas of the skill-scores used to evaluate the all-member ensemble mean and subsample mean rainfall predictions are summarized below:

The MSE skill (MSE-SS) is a measure of how the average accuracy of the forecasts compares to using a climatology alone (average estimate across a time series). MSE-SS is calculated by dividing the MSE_f, which is MSE (Equation 1) calculated for the forecasts, with the MSE_{clim}, which is MSE (Equation 1) calculated for the mean estimate across the time series (climatology mean over 1993-2016). This ratio is subtracted from 1, meaning a perfect score will result in 1, while a negative score means the MSE_f is worse than the MSE_{clim}.

$$\boldsymbol{MSE - SS} = \mathbf{1} - \frac{\boldsymbol{MSE_f}}{\boldsymbol{MSE_{clim}}} \qquad \text{Equation 4}$$

$MSE_f = MSE\ forecasts$
$MSE_{clim} = MSE\ climatology$

The Heidke Skill Score (HSS - Heidke, 1926) assesses a forecasts' ability to discriminate between different categories of events. In this study, HSS was applied to decile discrimination at the low

and high ends of the rainfall distribution, essentially testing the forecasts' ability to detect high rainfall extremes (HSS90 - an event is defined as rainfall above the 90th percentile of the monthly climatology) and low rainfall extremes (HSS10 - an event is defined as rainfall below the 10th percentile). Unlike other scores, like hit rate (correct detection rate of an event), or false-alarm rate (incorrect detection rate by predicting an event occurs when there is none), the HSS is equitable (Hogan et al., 2010), meaning random or rigged forecasts will all score 0.0 (no skill). As summarized by the following formula, HSS is calculated based on a 2x2 contingency table of hits (correct detections - a), false alarms (predictions of an event when there is none – b), misses (failures to detect an event – c), and correct rejections (not predicting an event will occur when there is none).

$$HSS = \frac{a+d-a_r-d_r}{n-a_r-d_r} \qquad \text{Equation 5}$$

$$a_r = (a+b)(a+c)/n$$
$$d_r = (b+d)(c+d)/n$$
$$a = correct\ detections$$
$$b = false\ alarms$$
$$c = misses$$
$$d = correct\ rejections$$
$$n = total\ number\ of\ instances$$

Text S2. Observational correlations that affect strength of subsampling method.

The R-squared values listed in the inset of Figure S1 provide a measure of how quickly the observations change on average between the reference period and the months in which the predictors are tested, which affects the strength of the subsampling method. These R-squared values show the correlation between the temporal mean of the predictors in last two weeks just prior to model initialization for a given forecast month, and the average monthly mean of the predictors in the two months following model initialization (one- and two- month leads). The Niño 3.4 region has the most stable SSTs compared to other predictors used and is more persistent during the September and October forecasting periods relative to earlier months (Figure S1 inset).

June is not an effective use case of the subsampling method, as this month is the least persistent observational forecasting period, with all observational correlations for wind direction equal to an R-squared of less than 0.1 and observational SST R-squared values below 0.5 for all zones tested (Figure S1 inset). Although the observed correlation between the reference data and two-month lead is typically worse than for one-month lead (Figure S1), two-month lead predictors were also tested as subsampling options because longer lead times give the ensemble members more time to diverge prior to evaluation.

Furthermore, although 925 hPA is at the peak intensity of the low level jets (Amador, 2008; Hidalgo et al., 2015), we used 850 hPA for wind direction because 850 hPA is available across all models evaluated and still falls within the range of the pressure levels of the low level jet streams. The model members were also consistently able to correctly identify the zonal wind direction within the maximum wind zone of the CLLJ (80W, 70W, 12.5N, 17.5N - Amador, 2008). There was more variability in the correct identification rate within the CJ and a broader zone that circumscribed the CLLJ and Central America, so we used a larger ‘Regional Wind’ box and CJ box as our final predictors to test how well the members identified zonal wind (Figure S1).

Text S3. Software

The subsamples were generated using XCast, a python software package that was developed to improve forecast post-processing and evaluation (https://github.com/kjhall01/xcast). The subsamples were processed using a combination of several other packages, including pysteps (Imhoff et al., 2023; Pulkkinen et al., 2019). We plotted all figures in R using tidyverse and ggplot2 (Huang & Zhao, 2020; Wickham, 2016; Wickham et al., 2019).

Text S4. Sensitivity test of subsample skill to ensemble size

We tested the sensitivity of the results to filter sizes in step four of the methodology, which refer to how many members are selected in each subsampling step. Figure S2 shows two examples of sensitivity tests run on the top-performing subsamples: (1) using different numbers of members to constrain the top subsample in May over Guatemala (using representation of TNA alone; Figure S2a), and (2) constraining the top subsample in September over Costa Rica (using representation of Niño3.4 at one-month lead, Niño3.4 at two-month lead, and Regional Wind at one-month lead; Figure S2b). Several studies have examined the effects of ensemble size on forecast skill (e.g. Buizza, 2008; Buizza et al., 1998; Buizza & Palmer, 1998; Leith, 1974; Leutbecher, 2019). Of these studies, many show increases in skill as size increases, but that the change in skill begins to saturate at ensemble sizes as small as eight (e.g. Buizza & Palmer, 1998; Leith, 1974). Similar to what these previous studies have shown, in the comparison in Figure S2, skill often declines abruptly under 10 ensemble members, but the added value of selecting more members that best represent key predictors do not dramatically increase when the number of ensembles is at/above ten in the final subsample.

The example using May forecasts in Guatemala shows the effects of changes in ensemble size alone (Figure S2a). The effect of selecting fewer ensemble members is illustrated most clearly when comparing MSE-SS (Figure S2a bottom row). Selecting members that best represent TNA at one-month lead begins to show an effect on skill using the top 30 members or less, and skill drops off between the top-10 and top-5 member subsamples.

When multiple predictors are used, the change in ensemble skill is often an indicator of sensitivity to a particular predictor over ensemble size (Figure S2b). For instance, in Costa Rica, filtering too strictly over Niño3.4 at one-month lead will limit skill (filtering by 60 members or less using Niño3.4 one-month lead representation in step 1 is associated with lower skill). Filtering more strictly for Niño3.4 at two-month lead is more useful (Figure S2b). Looking at differences within the top 80 members, for instance (left-hand side of Figure S2b), shows improved skill using the top 30 members that represent Niño3.4 SSTs at two-month lead over only selecting the top 40 members. In step three (still looking at left-hand side of S2b), filtering more strictly for representation of Regional Wind at one-month lead within the top 30 or 20 members often performs better up to the top 10 members (subsamples that use top 10/15 members for Regional Wind at one-month lead representation often have higher skill than selecting top 20 members that represent Regional Wind at one-month lead).

The top subsample for Costa Rica in September that is highlighted in the main article text was identified using HSS90 skill (significance between this subsample and the all-member ensemble is plotted in Figure S2b). This subsample represents a balance between filters, i.e. less strict filtering for the top members that represent Niño3.4 SSTs at one-month lead (top 80 members), more strict filtering for the top members that represent Niño3.4 SSTs at two-month lead (top 30 members), and additional filtering for the top 15 members that represent Regional Wind at one-month lead. While this subsample was highlighted in the main text, Figure S2b shows how filtering for the top 20 members that represent Niño3.4 SST at two-month lead in step 2 and the 10 members of Regional Wind at one-month lead in step three also performs well on multiple metrics.

Text S5. Sensitivity test of subsample skill to years analyzed

The all-member and subsampled anomaly predictions change year to year, and more closely follow the observations in some years over others (Figure S3). The subsampling method possibly has a greater effect on constraining the entire predicted range of the ensemble than changing the mean estimates (Figure S3). In May over Costa Rica (Figure S4 top row, left panel), the ensemble is the most constrained, as it only contains five members, whereas in other months and regions, the average percent change in the range of the predicted estimate is around 30-40% for subsamples that contain 10-15 members (Figure S3).

Although the constrained subsample range visually seems to follow the interannual pattern of the observations more closely than the all-member range, both forecasts completely miss the anomaly pattern in some years (e.g. May 2011 in Guatemala Figure S3 left panel bottom row). Because of these misses, we see opportunities to refine the approach beyond using a constant set of process-based criteria to generate subsamples and more flexibly emphasize different predictors for particular years (e.g. prioritize filtering members that represent Niño 3.4 SSTs in strong El Niño years), which may be related to the changing strength of predictors like ENSO and their relative importance to regional rainfall.

Although the bias varies interannually (Figure S3), our sensitivity analysis suggests the subsampling method is not necessarily sensitive to the time frame of analysis. Randomly dropping five years from the analysis has some effect on the skill of the all-member and top-subsample skill, but this does not often significantly affect the difference between the spread in skill of the all-member ensemble mean scores and the top subsample scores across each country (Figure S4). The difference in skill between the all-member and top subsample case for a given country and month often are still within the same level of significance (e.g. $p < 0.001$) regardless of whether the entire time-series (1993-2016) or a random subset that drops five years is used (significance tests plotted in Figure S4. Additionally, when a significance test was run between the 'All Years' and 'Drop 5' cases within each approach (e.g. between the top subsamples over Costa Rica), no significant differences were found between the boxplots except for MSESS in October (not plotted in S7 because N.S. occurs for all cases excluding October MSESS).

While year-to-year variability is worth investigating further to see how particular years may be more difficult to predict or, alternatively provide windows of opportunity for improved ensemble-based forecasts and subsampling methods, the average performance of the top subsamples is not likely dependent on performing well in key years alone. It would be worth continuing to test subsampled skill over more years outside of the 1993-2016 window, however, to see how the relevance of the different predictors maintains over larger time-periods.

To further operationalize the subsampling approach, next steps would be to identify top subsamples over a moving testing window, and then use a separate verification window to assess how long the top-subsample criteria maintains outside the window of analysis. This step could be combined with comparing different lengths of testing windows with consideration for potential years of interest (e.g. Strong El Niño periods) to determine optimal subsampling criteria prior to applying a subsampling approach in a real operational forecasting scenario.

Text S6. Average Model Representation in Subsamples

Subsample members come from different models depending on each models' performance in a given year. Models will vary from having zero representation in one year to making up more than 80% of the subsample in another, depending on their members' predictor scores. Table S2 highlights the average percentages of the members that are represented by a given model over the 24 year evaluation period (1993-2016) for each subsample type.

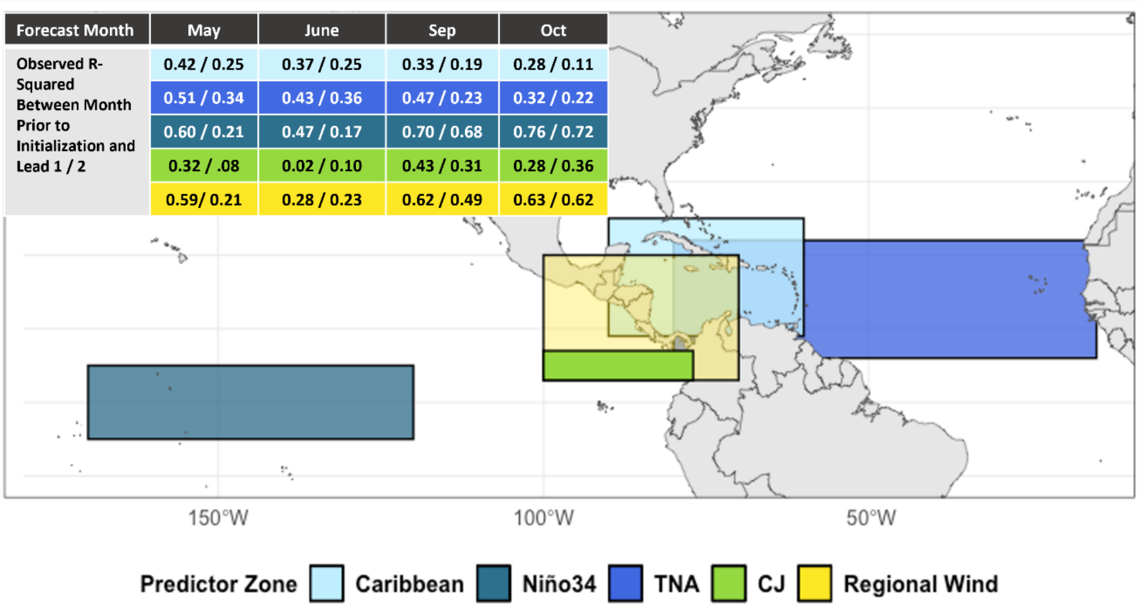

Forecast Month	May	June	Sep	Oct
Observed R-Squared Between Month Prior to Initialization and Lead 1 / 2	0.42 / 0.25	0.37 / 0.25	0.33 / 0.19	0.28 / 0.11
	0.51 / 0.34	0.43 / 0.36	0.47 / 0.23	0.32 / 0.22
	0.60 / 0.21	0.47 / 0.17	0.70 / 0.68	0.76 / 0.72
	0.32 / .08	0.02 / 0.10	0.43 / 0.31	0.28 / 0.36
	0.59/ 0.21	0.28 / 0.23	0.62 / 0.49	0.63 / 0.62

Figure S1. Predictor locations used to test member performance plotted spatially (same as Figure 1c) with an additional inset that includes the R-squared values of the correlation between the observed reference period and the predictor period for each prediction evaluated (Lead 1/Lead 2). Colors indicate predictor zone ordered by column of forecast month at two-month lead, May, June, September, and October.

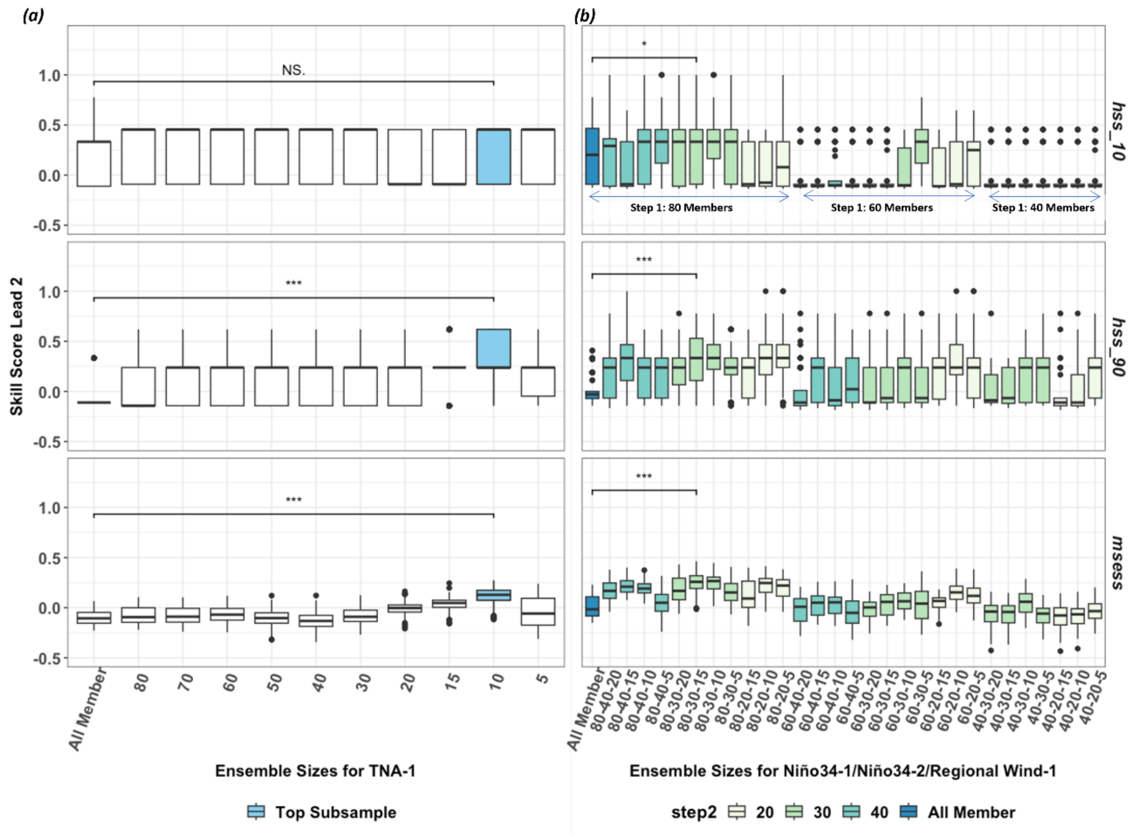

Figure S2. Assessing the effect of filter size on subsample skill. Comparisons are performed using boxplots with significance tests plotted (using a t-test) between the top-performing subsamples and the all-member mean ensemble for each skill score (HSS10 top row, HSS90 middle row, MSE-SS bottom row). *** indicate $p < 0.001$; ** indicate $p < 0.01$; * indicate $p < 0.05$. NS indicates non-significant. (a) Skill at lead two is plotted for Guatemala in May using different filter sizes for the top-performing predictor (TNA at one-month lead). Top subsample is highlighted in shaded box. (b) Skill at lead two is plotted for Costa Rica in September using different filter sizes for the top-performing predictor combination (Niño3.4 at one-month lead; Niño3.4 at two-month lead; Regional Wind at one-month lead). To clarify the comparison between step differences across multiple predictors, filter sizes are ordered by step 1 (80, 60, 40) as labelled in the top right box; followed by step 2 (40;30;20), which is shaded across box-plots, and finally by step 3.

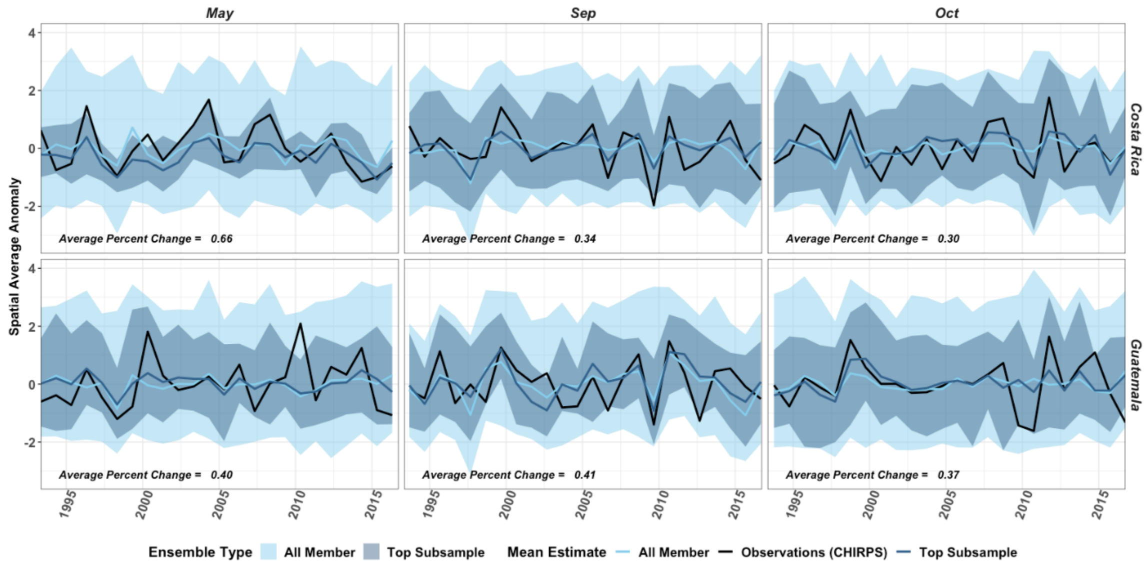

Figure S3. Comparing time series of the all-member and subsampled ensembles. Time series of spatially averaged total monthly rainfall anomalies for Costa Rica and Guatemala (rows) and three months (columns). Observed rainfall is shown in black (CHIRPS). The all-member ensemble (light blue) and best performing subsample (grey) are shown using the mean (lines) and range (maximum and minimum) of members. Table 1 in main article summarizes which criteria are used to generate the subsamples in each month/country. Average percent change is calculated for each panel to show the average difference between the predicted range of the all-member ensemble and the predicted range of the top subsample over the time series.

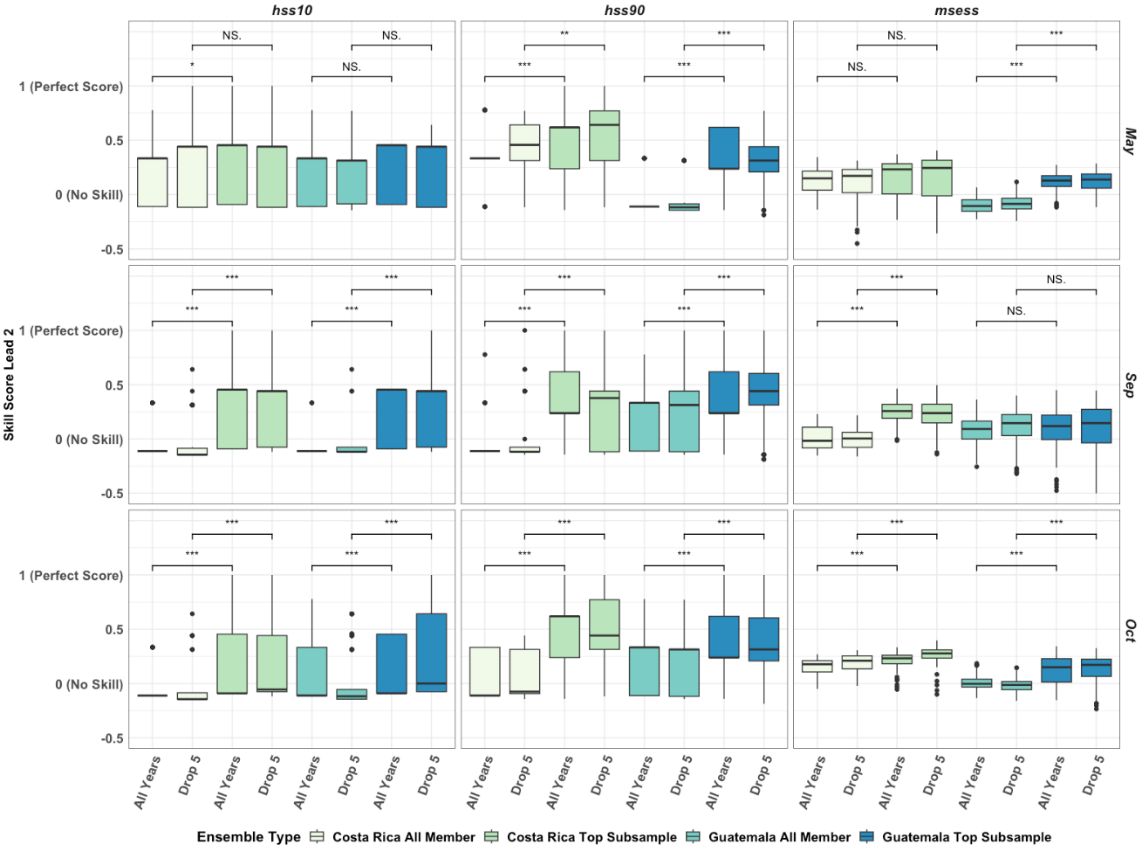

Figure S4. Sensitivity of skill to years analyzed. Skill comparison between the baseline, using all years in the analysis (1993-2016), and randomly dropping five years from the dataset. Boxplots show the spread in skill using HSS10, HSS90, and MSE-SS metrics (columns) across Costa Rica (left four boxplots for every panel) and Guatemala (right four boxplots for every panel) for the all-member and top subsamples for May, September, and October (rows). Significance tests using t-tests are run between the all-member mean scores and the top subsamples for both the 'All Years' and 'Drop 5' cases in both countries (4 tests per panel). *** indicate $p < 0.001$; ** indicate $p < 0.01$; * indicate $p < 0.05$. NS indicates non-significant.

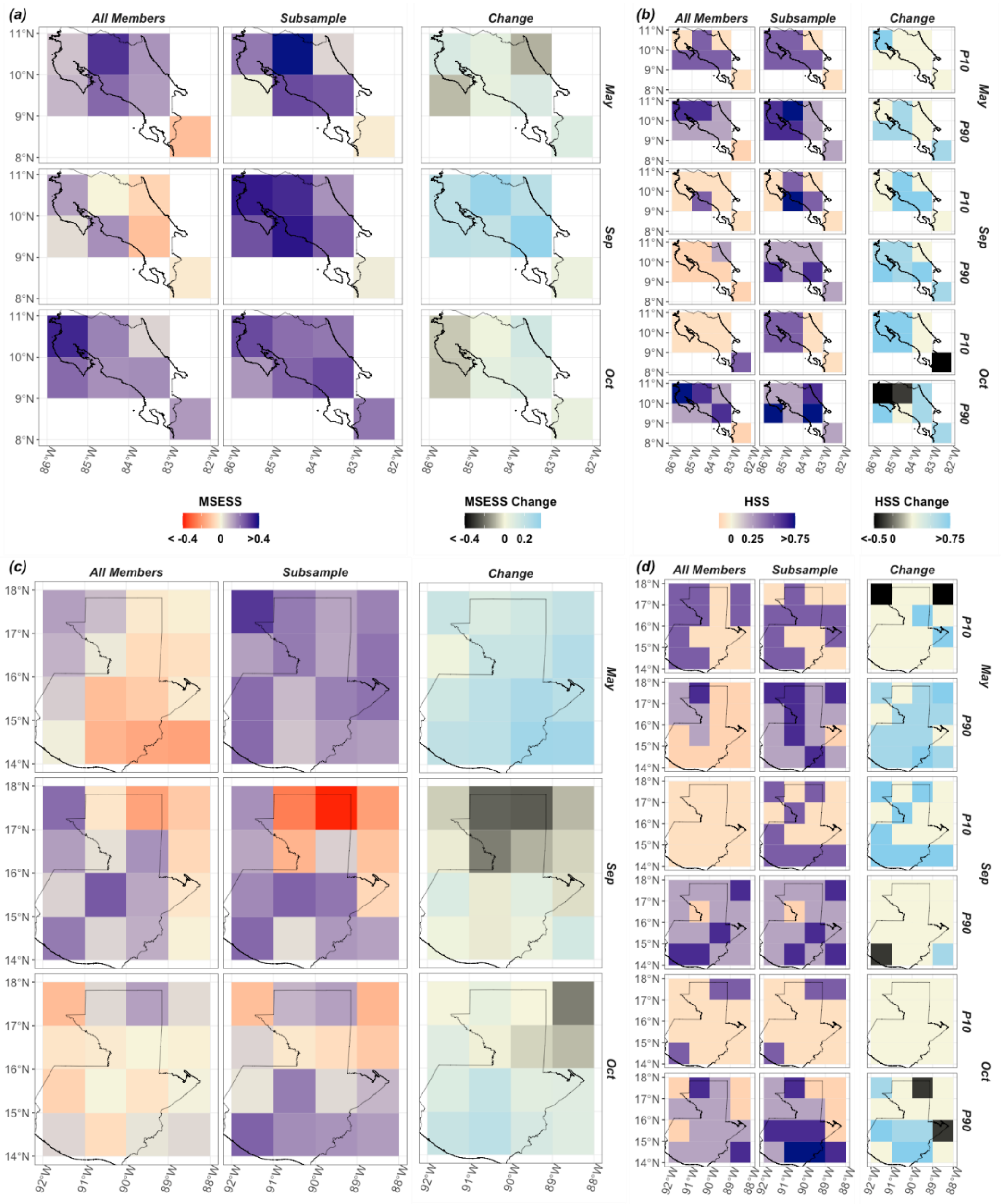

Figure S5. Spatial skill (same as Figure 3) but plotted at the raw 1° resolution where the CHIRPS observations are bilinearly regridded to the raw model spatial resolution before the point-wise skill is calculated. (a) Spatial skill of all-member ensemble (left), top performing subsampled ensemble (center) and difference between the two (right) in Costa Rica. Skill is based on the entire rainfall distribution using MSE-SS. (b) Spatial skill for detection of low rainfall extremes (P10) and high rainfall extremes (P90) using HSS in Costa Rica, again for all-member (left), subsample (center), and their difference (right). (c) Same as (a) but for Guatemala. (d) same as (b) but for Guatemala. Table 1 in main text summarizes the criteria used to generate the subsamples in each month.

Table S1. Summary of Models Used in Evaluation. Contributing center listed next to model name, total hindcast members used in analysis, and reference for the model. Abbreviations: CMCC, Fondazione Centro Euro-Mediterraneo Sui Cambiamenti Climatici; DWD, Deutscher Wetterdienst; ECCC, Environment and Climate Change Canada - Canadian Meteorological Centre; ECMWF, European Centre for Medium-Range Weather Forecasts.

Contributing Center	Model	Hindcast Members	Reference
CMCC	System 35	40	Gualdi et al. (2020)
DWD	System 2.1	30	Frölich et al. (2021)
ECCC	Cansips-IC3	10	Lin et al. (2021)
ECMWF	SEAS5	25	Johnson et al. (2018)
Meteo France	System 8	25	Batté et al. (2021)
Total Models in Analysis: 5 ; Total Members in Analysis: 130			

Table S2. Average Model Representation in Subsamples over the hindcast period (1993-2016). Criteria used to select top subsamples for each month correspond to the criteria in Table 1.

Month	Model Name (Total Members)	Average Model Member Representation over (1993-2016) (%)	
		Top Costa Rica Subsample	Top Guatemala Subsample
May	CMCC (40)	42%	35%
	DWD (30)	40%	24%
	ECCC (10)	3%	18%
	ECMWF (25)	11%	17%
	METEOFRANCE (25)	5%	7%
September	CMCC (40)	29%	29%
	DWD (30)	9%	18%
	ECCC (10)	3%	10%
	ECMWF (25)	30%	20%
	METEOFRANCE (25)	29%	23%
October	CMCC (40)	28%	22%
	DWD (30)	30%	28%
	ECCC (10)	8%	14%
	ECMWF (25)	14%	24%
	METEOFRANCE (25)	20%	13%
